# Self‐Organization of Long‐Lasting Human Endothelial Capillary‐Like Networks Guided by DLP Bioprinting

**DOI:** 10.1002/adhm.202302830

**Published:** 2024-02-20

**Authors:** Elsa Mazari‐Arrighi, Matthieu Lépine, Dmitry Ayollo, Lionel Faivre, Jérôme Larghero, François Chatelain, Alexandra Fuchs

**Affiliations:** ^1^ Université de Paris U976 HIPI, Inserm Paris F‐75006 France; ^2^ AP‐HP Hôpital Saint‐Louis 1 avenue Vellefaux Paris F‐75010 France; ^3^ CEA IRIG Grenoble F‐38000 France

**Keywords:** capillary networks, DLP bioprinting, tissue engineering, vascular engineering

## Abstract

Tissue engineering holds great promise for regenerative medicine, drug discovery, and as an alternative to animal models. However, as soon as the dimensions of engineered tissue exceed the diffusion limit of oxygen and nutriments, a necrotic core forms leading to irreversible damage. To overcome this constraint, the establishment of a functional perfusion network is essential. In this work, digital light processing bioprinting is used to encapsulate endothelial progenitor cells (EPCs) in 3D light‐cured hydrogel scaffolds to guide them toward vascular network formation. In these scaffolds, EPCs proliferate and self‐organize within a few days into branched tubular structures with predefined geometry, forming capillary‐like vascular tubes or trees of diameters in the range of 10 to 100 µm. Presenting a confluent monolayer wall of cells strongly connect by tight junctions around a central lumen‐like space, these structures can be microinjected with a fluorescent dye and are stable for several weeks in vitro. These endothelial structures can be recovered and manipulated in an alginate patch without altering their shape or viability. This approach opens new opportunities for future applications, such as stacking with other cell sheets or multicellular constructs to yield bioengineered tissue with higher complexity and functionality.

## Graphical abstract

Digital light processing bioprinting is used to guide primary endothelial progenitor cell self‐organization over light‐cured hydrogel scaffolds, producing capillary‐like branched vascular structures that are stable for weeks, with promising applications in tissue engineering.

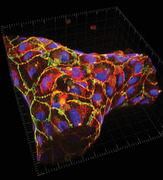

## 1 Introduction

Tissue engineering holds great promise in regenerative medicine, drug discovery, and as an alternative to animal models. Although tremendous effort has been invested so far, engineered tissue development is confronted with a major challenge. Once the multicellular entity reaches a certain size, a necrotic core occurs due to the lack of an efficient perfusion system to sustain cell survival.^[^ ^1^ ^]^ In vivo, blood vessels are essential for tissue homeostasis, supplying oxygen and nutrients, draining metabolic wastes, and transporting immune cells. These functions are enabled by a highly ramified vascular network formed by an inner monolayer of endothelial cells (ECs), overlain either by smooth muscle cells (SMCs) in large and medium‐sized vessels or by pericytes in capillaries.

For several years, many vascular tissue engineering strategies have been developed with the purpose of rebuilding or establishing a functional vascular system within an engineered tissue.^[^ ^2^ ^]^ In this regard, the decellularization of donor arteries or veins is highly valued for its ability to generate acellular scaffolds from native tissue, which may optionally be recellularized prior to implantation.^[^ ^3^, ^4^ ^]^ However, the need to find a suitable donor limits its use. As a result, a completely different line of research based on the creation of vascularized organoids from basic components and cells has been widely pursued. Through the differentiation of human pluripotent stem cells into EC and pericytes, Wimmer et al. developed a self‐organizing 3D vascular organoid capable of forming a stable and perfused vascular tree when transplanted into immunocompromised mice.^[^ ^5^ ^]^ Additionally, the differentiation of adult or induced stem cells has allowed the manufacture of various kinds of kidney, skin, or brain organoids composed of a vascular‐like network.^[^ ^6^, ^7^, ^8^ ^]^ As demonstrated with the recent development of an organoid model of diabetic vasculopathy,^[^ ^9^ ^]^ 3D culture and stem cell differentiation approaches offer a valuable alternative to animal models. Nevertheless, without appropriate spatial cues to guide cell self‐organization, this process remains highly stochastic, with no control over the size, accessibility, and 3D geometry of the vessels formed in the bulk of a hydrogel.

To address this challenge, several engineering strategies offer various levels of control over these criteria by guiding spontaneous cell self‐organization,^[^ ^10^ ^]^ including sheet‐based engineering^[^ ^11^, ^12^, ^13^ ^]^ and biocompatible polymer‐scaffolds approaches.^[^ ^14^, ^15^, ^16^, ^17^, ^18^ ^]^ Indeed, three‐layered vascular vessels of various diameters were manufactured by rolling a single cell sheet comprising a polydimethylsiloxane (PDMS) membrane that served as a support for human ECs, SMCs, and fibroblasts.^[^ ^19^ ^]^ Recently, bilayer vascular vessels of various diameters were obtained by co‐extrusion of a sacrificial polymer combined with hydrogels comprising human EC and SMCs,^[^ ^20^ ^]^ and by seeding both cell types onto a synthetic bilayer heparinized tubular structure created by combining electrospinning and freeze‐drying methods.^[^ ^21^ ^]^ However, with a resolution ranging from ≈100 µm to 2 mm in diameter, these technologies are unable to reproduce small vessels and more specifically capillaries (whose diameter varies from 20 µm down to a few micrometers). In vivo, capillaries form a complex and nevertheless, a well‐organized meshed network that ensures the proper supply of oxygen and nutriments to all tissues. To this end, adjacent capillaries are separated from each other by a maximum distance of 200 µm.^[^ ^22^ ^]^ In order to avoid the formation of an oxygen gradient, which might induce unforeseen cell differentiation or necrosis,^[^ ^1^, ^23^ ^]^ reproducing the capillary bed connected to a higher vascular tree therefore appears to be a key challenge for vascular tissue engineering.

Interestingly, digital light processing (DLP) is a promising 3D bioprinting technology that combines complete control over the 3D geometry at high resolution, while allowing high accessibility to the polymerized structure.^[^ ^24^ ^]^ Compared to other bioprinting techniques such as extrusion or inkjet which offer feature sizes of 150 to 400 µm,^[^ ^25^ ^]^ DLP offers unparalleled resolution down to 10 µl which is in the range of the smallest vessels of the vasculature. Using this approach, cells can be encapsulated in 3D photopolymerized hydrogels of the desired shape. By mimicking the mechanical properties of the natural cell environment,^[^ ^26^ ^]^ the 3D photopolymerized hydrogel also provides spatial cues to guide both cell proliferation and polarization. Using this method, we have previously manufactured a branched tubular epithelial network successfully mimicking the intrahepatic biliary tree.^[^ ^27^ ^]^

## 2 Results and Discussion

**Figure 1:**
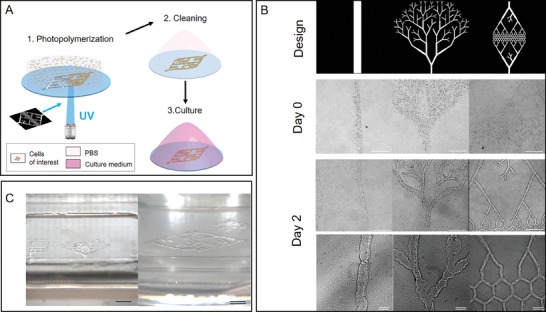
DLP photopolymerization of G^M^/C^M^/HA^M^/FG hydrogel structures encapsulating EPCs A) Principle of DLP bioprinting. B) Various digital patterns used to create photopolymerized structures embedding EPCs: a line, a branching tree, and a capillary bed‐type design, just after fabrication and 2 days after fabrication C) Sagittal view of EPC photopolymerized structures after 7 days of culture. Scale bars are 500 µm and double scale bars are 50 µm. C) Sagittal view of EPC photopolymerized structures after 7 days of culture. The scale bar is 2 mm and the double scale bar is 1 mm.

In this study, we used human endothelial progenitor cells (EPCs) from cord blood and DLP bioprinting to produce capillary‐like structures of controlled size and geometry by photopolymerization. ECs are known to spontaneously self‐organize into “cord‐like” structures when embedded in various hydrogel environments including collagen, gelatin, fibrin, and matrigel.^[^ ^28^, ^29^, ^30^, ^31^ ^]^ This self‐organization is highly disorganized, branching happens randomly, and structures rarely form stable lumens or vascular networks which could subsequently be manipulated and integrated into a bioengineered tissue construct. We thus developed a technique using DLP bioprinting^[^ ^24^ ^]^ to control the microenvironmental cues necessary to promote the self‐organization of EPCs into branched tubes with various sizes and shapes (**Figure** **1**A) using a similar experimental process.^[^ ^27^ ^]^ Briefly, a photopolymerizable mix of hydrogels, cells, and photoinitiator was placed between a glass coverslip and a PDMS roof, and UV light was projected as a DMD‐generated, mozaic image through the 10× objective of a microscope. Non‐polymerized hydrogel and cells were washed away and the resulting 3D structures were placed in the cell medium and cultured in a standard cell incubator for the indicated timeframes. Various DMD‐generated images and the resulting structures at day 0 and day 2 are shown in Figure 1B. These structures include a line, with the aim of creating a single tube with a given diameter; a tree, to explore the efficiency of creating a branching network as seen during angiogenesis; and a stylized capillary bed with line diameters going from 10 to 200 µm. Zoomed‐out images of the resulting constructs showing structures covering areas of 5 × 5 mm approximately are shown in Figure 1C.

A four‐component photopolymerizable hydrogel promotes the proliferation, migration, and self‐organization of EPCs into stable branched tubuloid networks with predefined size and geometry. Based on our previous success with epithelial biliary cells^[^ ^27^ ^]^ and on published studies using endothelial cells,^[^ ^32^ ^]^ we first explored various formulations incorporating either methacrylated gelatin (G^M^) or methacrylated type I collagen (C^M^) supplemented with fibrinogen (FG), methacrylated hyaluronic acid (HA^M^), and lithium phenyl‐2,4,6 trimethyl‐benzoyl phosphinate (LAP) – a cytocompatible photoinitiator^[^ ^33^ ^]^ (Figure 1B; Figure S1, Supporting Information). Indeed, G^M^ and C^M^ are photopolymerizable hydrogels produced from gelatin and collagen respectively which sustain good cell viability following encapsulation and retain natural cell‐binding motifs.^[^ ^34^, ^35^, ^36^ ^]^ HA^M^ is also a photopolymerizable hydrogel^[^ ^37^, ^38^ ^]^ derived from hyaluronic acid, which is found ubiquitously in native tissues and which is known to play an important role during angiogenesis^[^ ^39^ ^]^ and to be involved in endothelial cell proliferation and migration.^[^ ^40^ ^]^ FG is a component of the ECM that mediates functions such as adhesion, spreading, proliferation, and migration of a variety of cell types, including fibroblasts,^[^ ^41^ ^]^ epithelial,^[^ ^42^ ^]^ and endothelial cells.^[^ ^43^, ^44^ ^]^

We observed that one‐ and two‐component formulations failed to produce a tubular structure, unlike three‐component formulations which demonstrated encouraging results (Figure S1, Supporting Information). Indeed, EPCs encapsulated in G^M^/HA^M^/FG (2%:0.3%:0.4% in w/v) or C^M^/HA^M^/FG (0.2%:0.3%:0.4% in w/v) formulations showed active proliferation, migration and reorganization into tubular structures over the first few days in culture (see Movies S1 and S2 (Supporting Information), as well as Figure S1, Supporting Information). However, three‐component formulations also appear to be EPC donor‐dependent, with rapid degradation of the hydrogel in some cases (Figure S2, Supporting Information). This degradation was never observed in the absence of cells. We therefore hypothesized that our three‐component formulations failed to generate hydrogels strong enough to withstand the mobility of endothelial cells, and tested other approaches to overcome this limitation.

In the first approach, we increased the UV dose to increase chemical crosslinking and this indeed did lead to higher storage and loss moduli of the polymerized structures (Figure S3, Supporting Information).

However, we also observed increased cell death in these samples (Figure S4, Supporting Information) at day 1, from 60–70% viability at 10–20 mJ mm^−^^2^ down to 50–55% at 40 mJ mm^−^^2^, which is probably due to increased oxidative stress from the higher UV doses.

**Figure 2:**
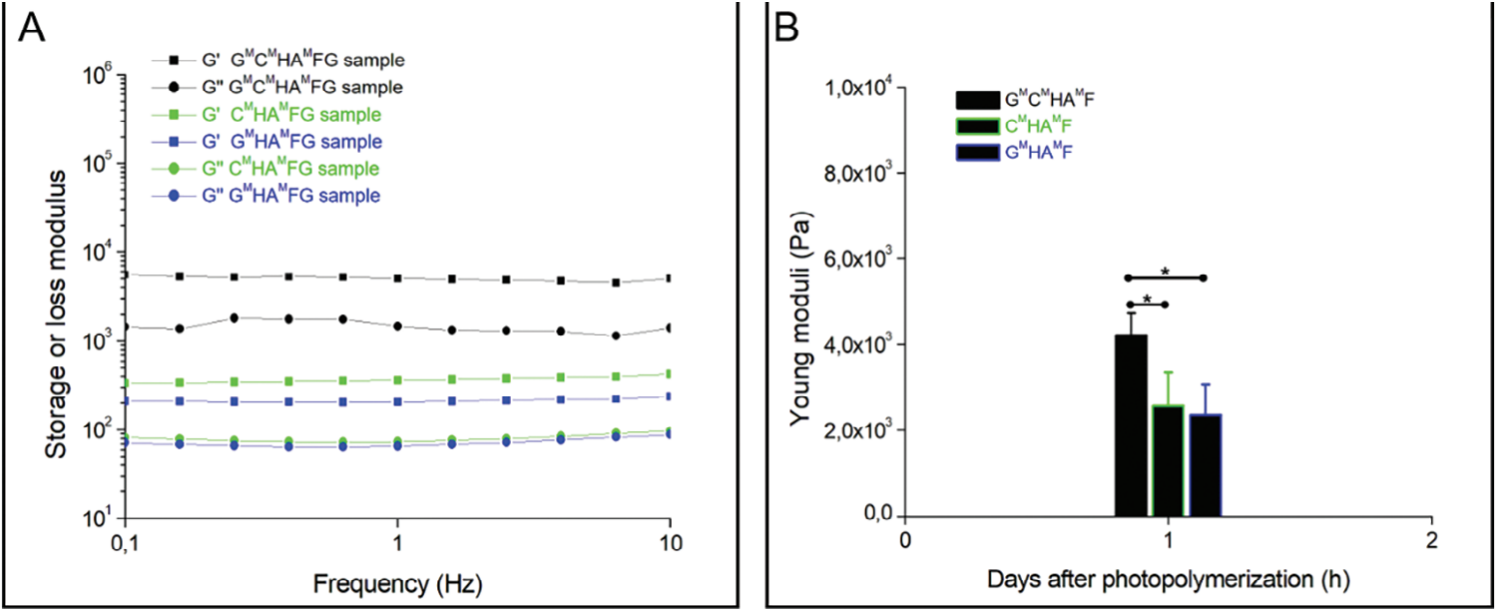
Rheological characterization of EPC encapsulated 3 different photopolymerized hydrogel structures using either frequency‐dependent oscillatory rheological analysis (A) or a compression test (B). Asterisks indicate significance with *p* < 0.05.

In a second approach, we screened higher concentrations of each hydrogel component, but were confronted to higher viscosity, heterogeneity, and less efficient washing of the structures after photopolymerization (data not shown). Finally, we studied a four‐component mix based on existing successful studies using a hybrid mix of both gelatin and collagen with endothelial cells.^[^ ^45^ ^]^ We thus mixed one volume of the gelatin formulation *G* ^M^ */HA* ^M^ */FG* with one volume of the collagen formulation *C* ^M^ */HA* ^M^ */FG* thus yielding the four‐component formulation: G^M^/C^M^/HA^M^/FG 1%:0.1%:0.3%:0.4% in w/v. We expected that given both three‐component formulations exhibited comparable G′, G″, and Y moduli (**Figure** **2**A,B), the four‐component mix would present similar mechanical properties. Surprisingly, this was not confirmed experimentally, and the four‐component mix showed a significant increase in both storage and loss modulus (Figure 2A), to reach values reported by Davidov et al.^[^ ^46^ ^]^ where they prepared an ECM matrix from decellularized arterial tissue which showed higher efficiency in generating stable angiogenic‐type growth in the standard in vitro assay. This significant increase in terms of stiffness was confirmed in compression tests with nearly a twofold increase in Young modulus from ≈2 kPa for the three‐component hydrogel to 4 kPa for the four‐component hydrogel (Figure 2B), also consistent with values reported by others.^[^ ^47^, ^48^ ^]^

**Figure 3:**
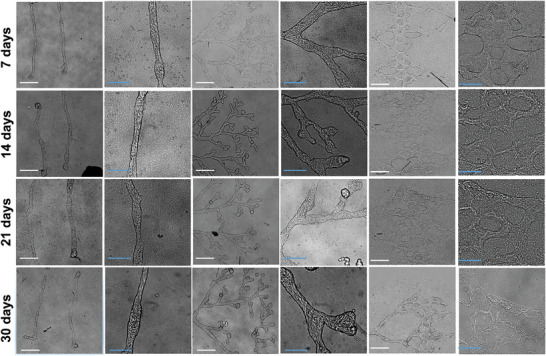
EPC photopolymerized hydrogel structures over time (between day 7 and day 30) Phase contrast images showing structures photo‐polymerized through a 10× objective at 20 mJ mm^−^^2^. Hydrogel formulations are composed of G^M^/C^M^/HA^M^/FG 1%:0.1%:0.3%:0.4% in w/v with 0.5% (wt vol^−1^) photoinitiator. Blue scale bars are 200 µm and white scale bars are 500 µm.

We also observed that the four‐component G^M^/C^M^/HA^M^/FG hydrogel supported higher cell viability during and after the photopolymerization process compared to either G^M^/HA^M^/FG or C^M^/HA^M^/FG (Figure S4, Supporting Information). More importantly, this hydrogel formulation showed substantially higher efficiency, reproducibility, and stability over time in forming tubuloids with considerable migration and reorganization of the EPCs within them (**Figures** 1 and **3**), and independently of the EPC donor (Figure S2, Supporting Information).

Finally, EPCs self‐organized with similar overall behavior and kinetics to form tubuloid‐type bodies of a wide diversity of shapes, branching, and sizes. Line structures from 10 to 200 µm in width and millimeters in length thus generated tubuloid structures of approximately the same width and length, whereas branched trees and honeycombed patterns (Figures 1, 3, as well as Movies [Link], [Link], Supporting Information) generated stable interwoven networks with good fidelity to the original design, spanning tens of mm^2^ (Figures 1, 3). The tree pattern was designed to mimic a developing capillary network, similar to what is observed during angiogenesis, whereas the honeycombed pattern with coalescing branches toward a single inlet and outlet on each side recapitulates the geometry of a stable capillary bed perfusing a tissue. All these tubuloid networks appeared to fully form and stabilize in ≈5–7 days and then remained stable in in vitro culture for up to 21 days and more (Figure 3).

**Figure 4:**
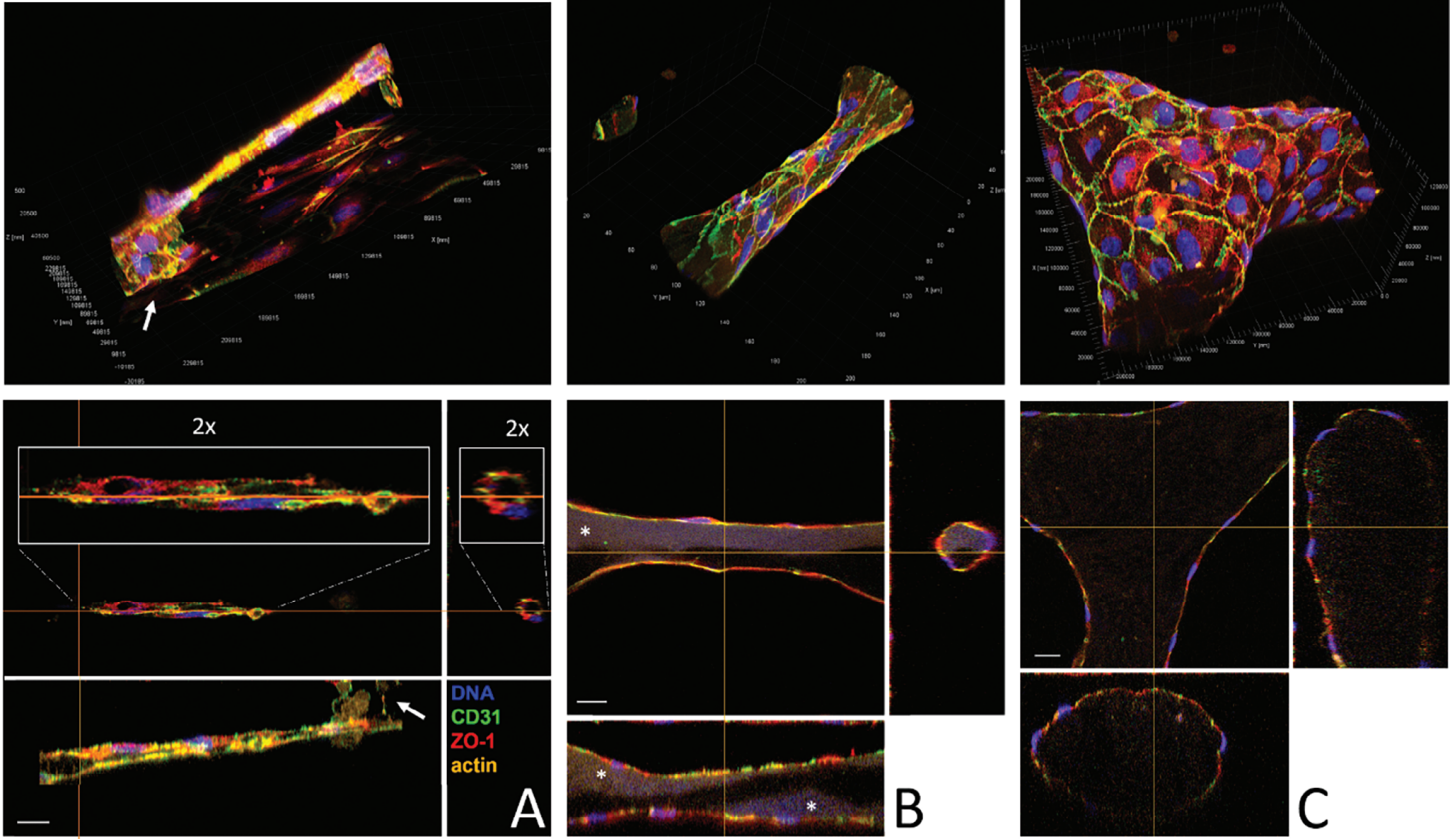
Characterization of EPC photopolymerized structures using confocal microscopy (day 7). Photo‐polymerized hydrogels are composed of the same 4‐component mix as above. Confocal images and orthogonal views show structures photo‐polymerized through a 10× objective at 20 mJ mm^−^^2^ after 7 days of culture in A) a 10 µm line, B) a 50 µm line, and in C) at a branching point of a tree. Autofluorescence of the collagen is indicated with a * and anchoring points of the 10 µm tube onto the glass slide are indicated with white arrows. 2× inserts correspond to zoomed areas on the sections of the 10 µm tube. The scale bar is 20 µm.

After 7 days in culture, tubuloid structures developed a tight monolayer wall of endothelial cells spanning a central single lumen‐like space, mimicking the cell organization of a blood capillary. Confocal microscopy was used to characterize the internal cell organization of the tubuloid structures (**Figure** **4**). Labeling of *F*‐actin, nuclei, tight junctions, and of CD31, specific for endothelial cells, showed that these tubuloid structures were exquisitely mimicking capillary endothelial tubes with a tight monolayer of confluent cells forming a circumferential wall around a central space devoid of cells. In certain areas, this tube was observed anchored at a point on the glass coverslip (depicted in Figure 4A on a 10 µm tube with white arrows) and then rising a few tens of microns above the glass coverslip plane on which a standard 2D layer of endothelial cells was also visible. Intense ZO1 and CD31 labeling largely concentrated at the junctions between adjacent cells and showed no defects in the wall. As depicted in Figure 4C, branching points imaged from tree‐designs also exhibited a full external monolayer wall with a continuous Y‐shaped cavity. The endothelial constructs were approximately the same diameters as the original photopolymerized hydrogel structures as seen in Figure 4A,B which show respectively tubes obtained with 10 and 50 µm hydrogel lines. The faint fluorescent signal observed inside the structures was attributed to the typical autofluorescence of collagen (indicated by *), demonstrating that the initial hydrogel remained present to a certain extent. Interesting, darker patches inside the structures could also be observed, sometimes spanning hundreds of microns over half the section of the tube (Figure 4B). This could therefore point to a partial degradation of this hydrogel scaffold over time. Interestingly, in these areas, the single monolayer cell wall did not appear to collapse, suggesting a different type of support, either linked to some osmotic pressure or the presence of a newly synthesized extracellular matrix which would need further in‐depth studies to elucidate.

**Figure 5:**
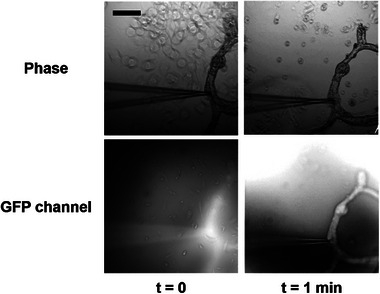
Microinjection of EPC tubes at 7 days of culture. Before perfusion, the microinjection pipet was inserted into the endothelial tube while the opposite side of the EPC tube was cut. The scale bar is 100 µm.

Endothelial tubes can be microinjected with a fluorescent dye. A pulled glass capillary tip linked to a standard microinjection setup was positioned in the internal space of a seven‐day‐old branched network or tube as depicted in **Figure** **5** under a baseline positive pressure of 35 hPa to avoid clogging the pipette. An injection at 150 hPa during one second was then applied to inject the fluorescein. We found that fluorescence could be detected in the endothelial tube only if the tube was physically opened at a distal end (outside the camera view). In this case, despite a high fluorescence background linked to the fluorescein dye leaking out, a high level of fluorescence is observed inside the tube and fluorescein rapidly diffuses along the tubular network in less than a minute as illustrated in Figure 5, demonstrating that the internal space is uninterrupted even across multiple branching points.

**Figure 6:**
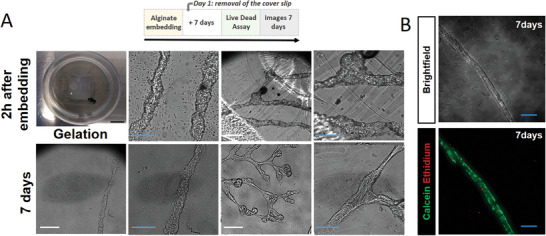
Images representing EPC structures (A) and cell viability (B), 7 days after embedding in an alginate patch. In the Live/Dead Assay (B), live cells are labeled by calcein (green) and dead cells by ethidium (red). Blue scale bars are 200 µm and white scale bars are 500 µm.

Endothelial networks can be removed from the coverslip surface and manipulated as a flexible soft cell sheet for further tissue engineering applications. With the aim of integrating these highly organized endothelial networks with other bioengineered cell constructs and in particular with the aim of creating transplantable tissue, it became necessary to develop a method to remove the structures from the supporting glass coverslip without damaging them. As embedding cells in alginate hydrogels (as beads, fibers, or sheets) is a common strategy to preserve cell function and viability^[^ ^49^, ^50^, ^51^ ^]^ while offering a convenient matrix for further manual handling, we explored various approaches and found that depositing a drop of 1.5% alginate inside a silicone frame surrounding the construct and then letting it gel by immersing it into a calcium solution was efficient in embedding the endothelial structures in a thin layer. After transfer into the cell culture medium, the thin sheet of alginate spontaneously delaminated from the glass chip, peeling away the endothelial structures and thus providing an easy and reproducible way to recover and manipulate mature endothelial structures while preserving their shape and viability (**Figure** **6**).

## 3 Conclusion

Endothelial cells are known to spontaneously and rapidly form cord‐like structures when plated in a basal membrane extract in a medium containing angiogenic factors. This propensity is used as a functionality readout in what is known as an endothelial tube formation assay, a widely used method for studying in vitro angiogenesis. Tube formation is sustained for 18–24 h, and lumens may randomly form along the cords, after which time the tube networks disintegrate. The addition of pericytes^[^ ^52^ ^]^ or mesenchymal stem cells^[^ ^53^ ^]^ has been shown to stabilize the formation of endothelial luminal tubes in 3D co‐culture conditions. We show here that not only can we create long‐lasting networks of endothelial tubes from endothelial cells alone, but we can also build linear tubes spanning millimeters, as well as branched networks mimicking a microvascular bed according to a predetermined design. This work thus illustrates how engineering approaches, in particular gel micropatterning as well as bioprinting, can guide cellular self‐organization into building reproducible functional tissue entities and mini‐organs with high fidelity and robustness, as discussed in our review,^[^ ^10^ ^]^ and demonstrated experimentally in recent advances by our team and other labs.^[^ ^27^, ^37^, ^38^, ^54^, ^55^ ^]^ Compared to our previous study with biliary tubes,^[^ ^27^ ^]^ endothelial cells appeared to require a stiffer environment to self‐organize into stable tubes and we therefore had to resort to a more complex hydrogel mix associating both gelatin and collagen I, with hyaluronic acid and fibrin to reach a formulation that consistently showed efficiency in forming stable networks with EPCs from various donors.

Our approach shows in particular great potential in tissue engineering applications. Indeed, as no bulk hydrogel is present around the structures, the endothelial structures obtained in this setup offer free access to the outside of the tube, allowing in follow‐up studies the addition of other cell types, such as SMCs and pericytes, at any stage of the vascular bed maturation, to obtain more complex and physiological blood vessel models. What's more, by demonstrating that we can recover the structures in a soft patch and manipulate it as a sheet, we can envisage follow‐up studies where we would integrate this sheet with other prefabricated tissues and organoids by essentially stacking the thin sheets one on top of the other. This however is no trivial feat as one is immediately confronted to issues of optimizing cell medium composition to accommodate the needs of all cell types that are present.

Perfusion of this vascular model in vitro remains however a challenge. Even though we show the first successful attempts at microinjecting fluorescent dyes into the tubular network, developing a robust method to connect a microfluidic system to the inlet and outlet ports of such a network to bring nutrients and oxygen to the bioengineered tissue, and recapitulate physiological shear stress, still needs to be addressed. As the 4‐component hydrogel formulation seems to partially degrade over time, it would also be interesting to elucidate if the cells are replacing this biodegradable scaffold with newly synthesized ECM, either on the apical side or basal side, and if the addition of pericytes may further contribute to the formation, maintenance, and remodeling of a basement membrane^[^ ^52^, ^56^ ^]^

## 4 Experimental Section

### Cell Sources and Reagents

Endothelial progenitor cells (EPCs) – EPCs were collected from two independent donors by isolating mononuclear cells from human umbilical cord blood according to the previously published protocol.^[^ ^57^ ^]^ Healthy donors’ cord blood was obtained from Saint‐Louis Hospital Cord Blood Bank (French Ministry of Research registration number AC‐2016‐2756 and French Normalization Agency number 201/51 848.1).

EPCs were cultured in EGM‐2 (Lonza) under 5% CO_2_ at 37 °C. Only cells from passages 2–6 were used in the following experiments. All reagents were obtained from Sigma Aldrich, France unless otherwise specified.

### Fabrication of Hydrogel Structures Encapsulating Ecs Using Dlp Bioprinting

Photopolymerizable hydrogel formulations were prepared in PBS with final concentrations of 1% or 2% (w/v) methacrylated gelatin (G^M^), 0.1% or 0.2% (w/v) type I methacrylated collagen (C^M^, Advanced BioMatrix), 0.3% (w/v) methacrylated hyaluronic acid (HA^M^, Advanced BioMatrix), 0.4% (w/v) fibrinogen (FG), and 0.5% (w/v) lithium phenyl‐2,4,6 trimethyl‐benzoyl phosphinate (LAP, Allevi). ECs were harvested with 0.25% trypsin‐EDTA and the cell pellet was resuspended at a cell density of 6 × 10^6 ^cells mL^−1^ directly in the photopolymerizable hydrogel formulation.

The DLP setup is described in the previous work.^[^ ^27^ ^]^ A volume of 35 µL of the photopolymerizable hydrogel formulation containing 6 × 10^6^ cells mL^−1^ was dispensed into the space between a methacrylated glass coverslip and a PDMS pad, separated by a silicone spacer of 250 µm height (SIN 400 T, Sterne, France). To build the single‐layer scaffold, UV light was projected through the 10× objective of the microscope according to the chosen optical photopattern for a calibrated curing time to reach a dose of 20 mJ mm^−^^2^ unless stated otherwise. The system corresponded roughly to an exposure of 4 s for each mm^2^. After UV exposure, the PDMS stamp was removed and the structures were washed with PBS to remove the unpolymerized fraction of the hydrogel/cell formulation. The glass coverslip bearing the hydrogel/cell structures was transferred to a cell culture dish and cultured in an EGM‐2 culture medium. The culture medium was renewed 3 times a week.

### Cell Viability Assays

Each previously described hydrogel mix was gently mixed with EPCs at a cell concentration of 6 × 10^6^ EPCs mL^−1^ and was photopolymerized with various UV doses: 10, 20, 40, and 60 mJ mm^−^^2^ doses. Cell viability was checked, 4 and 24 h after photopolymerization respectively, by staining with a live and dead assay kit (Live/Dead Cell Viability Assay, Thermo Fisher Scientific) following the manufacturer's manual.

### Fixation, Immunofluorescent Staining, and Confocal Microscopy

To investigate the endothelium formation in vitro, encapsulated EPCs in photopolymerized hydrogel structures were cultured for a week in an EGM‐2 culture medium and then fixed, labeled, and imaged as described previously.^[^ ^27^ ^]^ The following, was the list of used antibodies and dyes with the dilution ratios relative to stock concentrations recommended by the manufacturers: anti‐CD31 (Abcam #ab28364) – 1:100; anti‐ZO‐1 (Invitrogen #33–9100) – 1:100; goat anti‐Mouse IgG (Cy3) (Abcam #ab97035) – 1:200; goat anti‐Rabbit IgG (Alexa Fluor 488) (Thermo Fisher Scientific #A‐11034) – 1:200; phalloidin (Alexa Fluor 647) (Thermo Fisher Scientific #A22287) – 1:200; DAPI (Roche #10 236 276 001) – 4 µg mL^−1^.

### Time‐Lapse Image Acquisitions

Time‐lapse microscopy was performed at 37 °C and 5% of CO2, with images taken at 30 min intervals using a Leica DMi8 microscope equipped with a 10× objective.

### Rheological Analysis

The rheological features were evaluated for all three different photopolymerized hydrogel formulations: G^M^/HA^M^/FG 2%:0.3%:0.4% (w/v), C^M^/HA^M^/FG 0.2%:0.3%:0.4% (w/v) and G^M^/C^M^/HA^M^/FG 1%:0.1%:0.3%:0.4% (w/v).

Oscillatory measurements: Storage and loss moduli of photopolymerized hydrogels were measured by a Discovery HR 2 rheometer (TA Instruments) equipped with a parallel plate geometry (*d* = 25 mm). Samples with a thickness of 250 µm and 25 mm in diameter were photopolymerized with either a 20 mJ mm^−^^2^ dose or a 40 mJ mm^−^^2^ dose. The photopolymerized samples were subjected to oscillatory measurements after being incubated in an EGM‐2 medium for 1 day at 37 °C. The frequency sweeps were conducted at room temperature in the range of 0.1–10 Hz, and with a constant strain rate of 2%, considered to be in the linear viscoelastic range.

Compression tests: The elastic modulus of the resulting photopolymerized hydrogels was determined on a Microtester G2 (CellScale) on rectangular pads with a thickness of 250 µm and 6 mm^2^ area at room temperature. Before testing, the samples were incubated for 1 day in EGM‐2 at 37 °C to reach swelling equilibrium. The compression test was conducted at 10–20% strain with 2 µm s^−1^ strain rate. The elastic modulus of each sample was calculated from the linear region of the stress–strain curve using Origin software with the force and displacement data collected from the Cell Scale software.

### Microinjection Setup

The micropipette used for the microinjection of vascular tubes was made from borosilicate glass tubes with an outer diameter of 1.2 mm and an inner diameter of 0.94 mm (Harvard Apparatus). The injection micropipette was pulled using a micropipette puller (PC‐100, NARISHIGE). The resulting injection micropipette was backfilled with 5–10 µL of 500 µg mL^−1^ fluorescein solution and was mounted on the microinjection manipulator (InjectMan 4, Eppendorf) connected to a pneumatic microinjection pump (FemtoJet 4i, Eppendorf) with a compensation pressure of 35 hPa. The injection micropipette was introduced in the endothelial tube on one end of the targeted tubular network while the distal end was cut using surgical scissors. Once the tip of the micropipette was positioned within the tube of the EPC photopolymerized structures, the fluorescein solution was injected at 150 hPa during 1 s and was tracked under an inverted microscope (Nikon Ti2, Nikon) using a 10× objective.

### Encapsulation of Endothelial Networks Within an Alginate Layer

After removing the culture medium, a drop of 50 µL of 1.5% Na‐alginate (Wako PureChemical Industries) was placed over the endothelial structures on the glass coverslip inside a 1 cm^2^ frame cut out from a 250 µm thick silicone sheet (SIN 400 T, Sterne, France). This allowed the drop to spread out evenly in a thin film over the structures. The glass coverslip was then gently immersed into a mixture of 100 mm CaCl_2_ and 3% w/w sucrose solution for 10 min. The CaCl_2_/sucrose solution was removed and replaced by an EGM‐2 culture medium. The alginate layer spontaneously detached from the glass coverslip after 15–30 min, and was transferred into a 35 mm petri dish and cultured in the EGM‐2 under 5% CO_2_ at 37 °C.

### Statistical Analysis

Data were processed with Origin software (Origin Lab Inc). Each experiment was carried out with at least four individual samples. All the values were given as the mean ± standard deviation. Statistical analysis was performed using Student´s *t*‐test, with *P *= 0.05 considered to be statistically significant. For viability assays in Figure S4 (Supporting Information), it was also assessed for the significance of the differences between groups by using a one‐way analysis of variance with *P *= 0.05 considered to be statistically significant.

## Conflict of Interest

The authors declare no conflict of interest.

## Supporting information

Supporting Information

Supplemental Movie 1

Supplemental Movie 2

## Data Availability

The data that support the findings of this study are available from the corresponding author upon reasonable request.
