# Supplementary material for: Self‐Organization of Long‐Lasting Human Endothelial Capillary‐Like Networks Guided by DLP Bioprinting
Source: Adv Healthc Mater. 2024 Feb 20;13(14):2302830. doi: 10.1002/adhm.202302830 (PMC11468676; doi:10.1002/adhm.202302830)
Supplement: Supplementary file 1 — Supporting Information [file ADHM-13-2302830-s001.pdf]

ADVANCED HEALTHCARE MATERIALS

Supporting Information

for *Adv. Healthcare Mater.*, DOI 10.1002/adhm.202302830

Self-Organization of Long-Lasting Human Endothelial Capillary-Like Networks Guided by DLP Bioprinting

*Elsa Mazari-Arrighi, Matthieu Lépine, Dmitry Ayollo, Lionel Faivre, Jérôme Larghero, François Chatelain and Alexandra Fuchs**

SUPPLEMENTAL INFORMATION

Self-organization of Long-lasting Human Endothelial Capillary Networks Guided by DLP Bioprinting

Elsa Mazari-Arrighi, Matthieu Lépine, Dmitry Ayollo, Lionel Faivre, Jérôme Larghero, François Chatelain, Alexandra Fuchs*

Affiliations:

Université de Paris, Inserm, U976 HIPI, F-75006, Paris, France.

E. Mazari-Arrighi, M. Lépine, D. Ayollo, L. Faivre, J. Larghero, F. Chatelain, A. Fuchs

AP-HP, Hôpital Saint-Louis, 1 avenue Vellefaux F-75010, Paris, France.

E. Mazari-Arrighi, M. Lépine, D. Ayollo, L. Faivre, J. Larghero

CEA, IRIG, F-38000, Grenoble, France.

F. Chatelain, A. Fuchs

*Corresponding author:

Alexandra Fuchs alexandra.fuchs@cea.fr

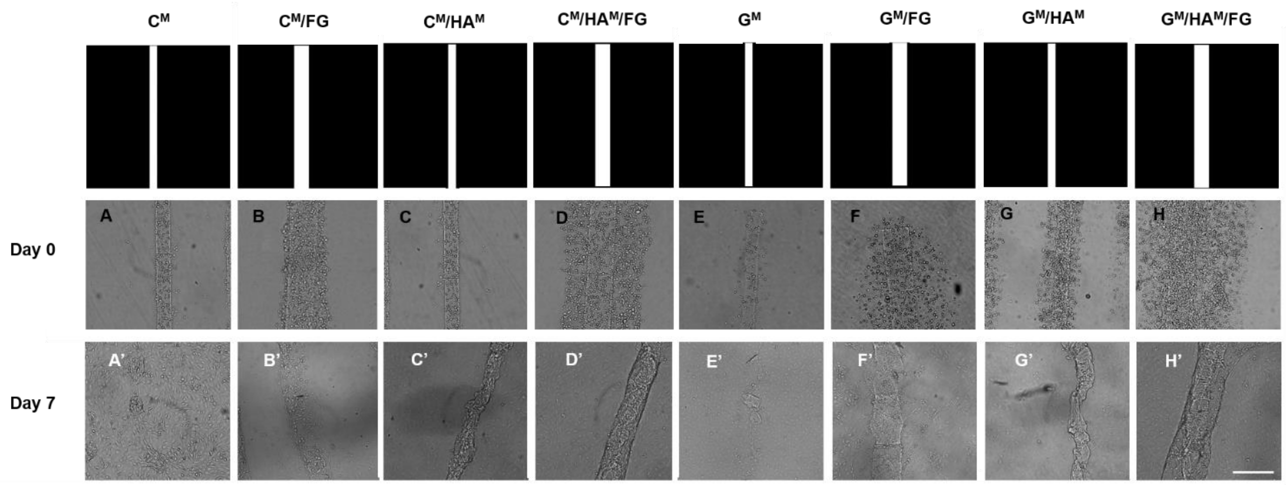

Figure S1: Culture over time of EPCs photopolymerized in one-component, two-component, and three-component hydrogel structures. Optical images showing EPCs encapsulated in photopolymerized hydrogels (A-H) just after photopolymerization and (A'-H') 7 days after photopolymerization. EPCs were embedded respectively in (A-A') C^M, (B-B) mixed C^M and FG, (C-C) mixed C^M and HA^M, (D-D') mixed C^M and HA^M and FG, (E-E') G^M, (F-F') mixed G^M and FG, (G-G') mixed G^M and HA^M, (H-H') mixed G^M and HA^M and FG. C^M hydrogel is mixed at 0.2 % (w/v) ratio, C^M/FG is mixed at 0.2 % (w/v):0.4 % (w/v) ratio, C^M/HA^M is mixed at 0.2 % (w/v): 0.3 % (w/v) ratio, and C^M/HA^M/FG hydrogel is mixed at 0.2 % (w/v):0.3 % (w/v):0.4 % (w/v) ratio. G^M hydrogel is mixed at 2 % (w/v) ratio, G^M/FG is mixed at 2 % (w/v):0.4 % (w/v) ratio, G^M/HA^M is mixed at 2 % (w/v): 0.3 % (w/v) ratio, and G^M/HA^M/FG hydrogel is mixed at 2 % (w/v):0.3 % (w/v):0.4 % (w/v) ratio. LAP concentration is 0.5 % (w/v) for all experiments. Applied UV doses are 20 mJ.mm^{-2}. Scale bar is 100 µm.

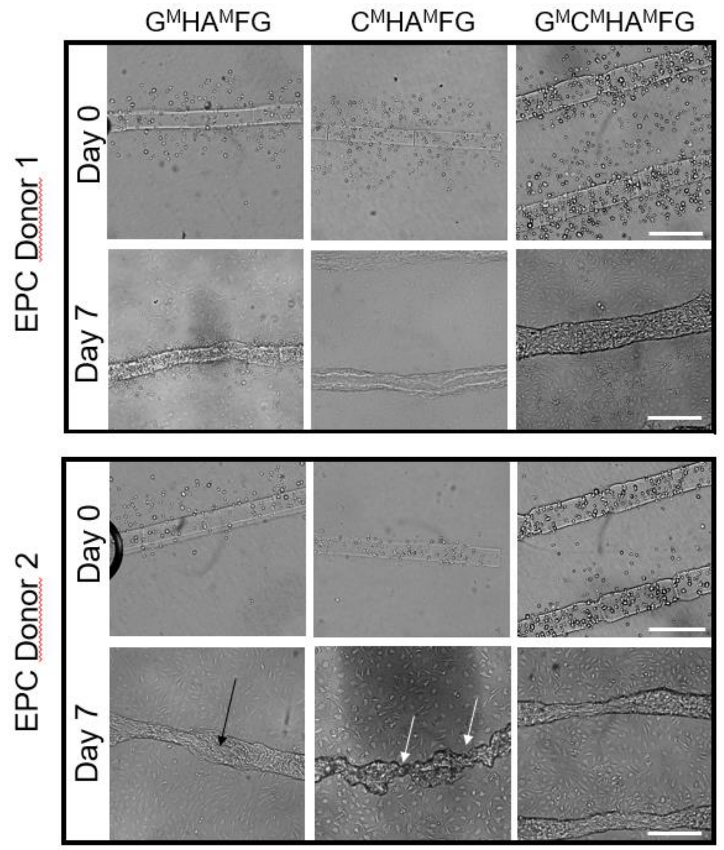

Figure S2: Phase contrast images showing the heterogeneity of tubular formation between EPCs isolated from different donors. EPCs were encapsulated by photopolymerization through a 10X objective at 20 mJ.mm-^{2} in GM/HAM/FG, CM/HAM/FG or GM/ CM/HAM/FG hydrogels. Arrows indicate construct either partially (black) or completely (white) collapsed. Scale bars are 200 µm.

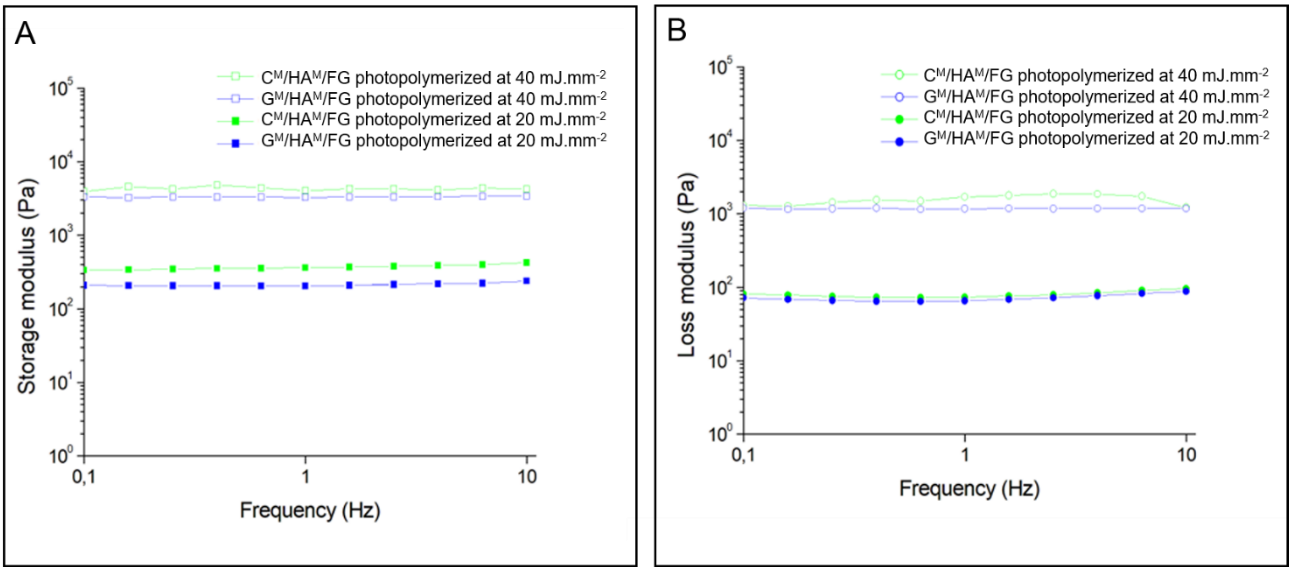

Figure S3: Rheological characterizations of three-component photopolymerized hydrogel formulations using frequency-dependent oscillatory rheological analysis as a function of UV dose. Resulting (A) storage moduli. (B) loss moduli.

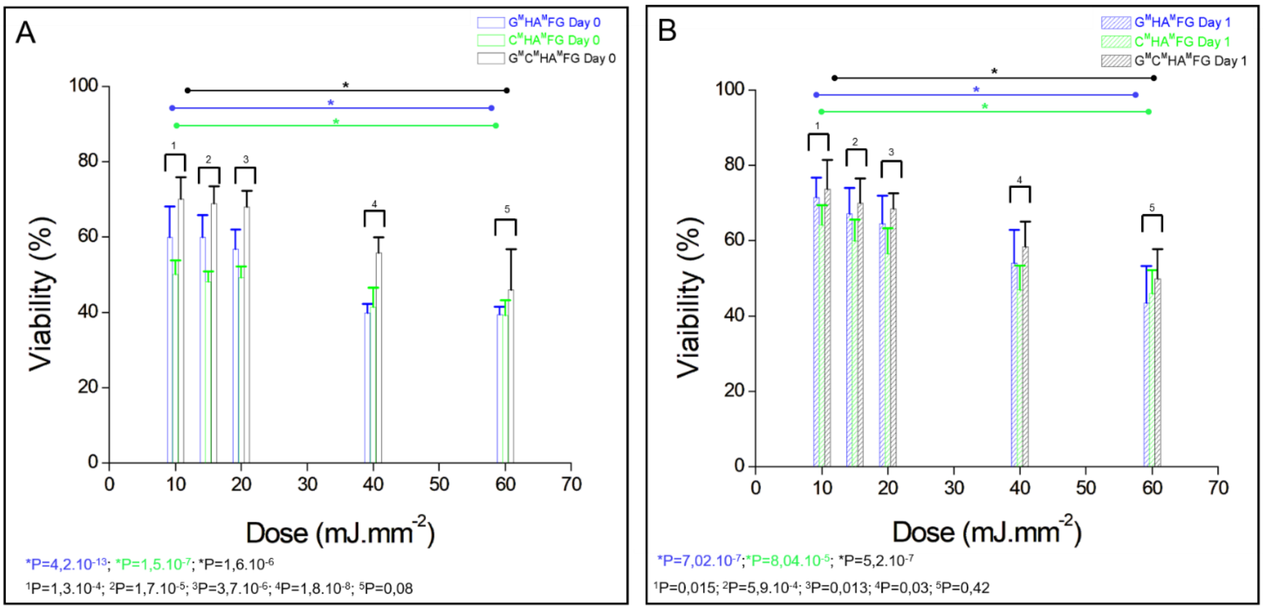

Figure S4: Bar chart showing cell viability of EPCs encapsulated within 3 different photopolymerized hydrogel structures 4 h (A) and 24 h (B) after the photopolymerization process. G^M/HA^M/FG hydrogels were mixed at 2 % (w/v):0.3 % (w/v):0.4 % (w/v) ratio, C^M/HA^M/FG hydrogels were mixed at 0.2 % (w/v):0.3 % (w/v):0.4 % (w/v) ratio, and G^M/C^M/HA^M/FG hydrogels were mixed at 1 % (w/v): 0.1 % (w/v): 0.3 % (w/v):0.4 % (w/v) ratio. LAP concentration is 0.5 % (w/v) for all experiments. Error bars represent ± SD and n = 4 at least for all data points. The differences between the viability means of EPC cured with a respective UV dose of 10, 15, 20, 40, and 60 mJ.mm^{-2} within a specific photopolymerized structure (either G^M/HA^M/FG, C^M/HA^M/FG, or G^M/C^M/HA^M/FG) were assessed by using analysis of variance (ANOVA). At the 0.05 level, 4 h and 24 h viability means exhibited by EPC cured at 10, 15, 20, 40, and 60 mJ.mm^{-2} UV doses were significantly different from each other for the G^M/HA^M/FG photopolymerized hydrogel mix, for the C^M/HA^M/FG one, and for the G^M/C^M/HA^M/FG one. The corresponding p-values are indicated with the blue, green, and black asterisk. ANOVA was also employed to compare the viability means of EPC encapsulated within G^M/HA^M/FG, C^M/HA^M/FG, and G^M/C^M/HA^M/FG photopolymerized hydrogel structures at a specific UV dose (either 10, 15, 20, 40, or 60 mJ.mm^{-2}). At the 0.05 level, viability means exhibited by EPC encapsulated within G^M/HA^M/FG, C^M/HA^M/FG, and G^M/C^M/HA^M/FG photopolymerized hydrogel structures were significantly different from each other when cured with a 10, 15, 20, and 40 mJ.mm^{-2} UV dose, 4 h and 24 h after the photopolymerization process. Conversely, the viability means obtained for EPC embedded within G^M/HA^M/FG, C^M/HA^M/FG, and G^M/C^M/HA^M/FG photopolymerized hydrogel structures were not significantly different at the 0.05 level when crosslinked with a 60 mJ.mm^{-2} UV dose, 4 h and 24 h after the photopolymerization process. Each p-value for each UV dose is stated by a number.

Movies:

M1_50u.avi & M2_Tree.avi : Timelapse acquisitions of EPCs embedded in a line-shaped hydrogel pattern of 50 µm width (50u.avi) or in a tree-shaped hydrogel pattern (Tree.avi) over 60 hours in

culture. Phase contrast images were captured every 30 min starting 24 h after photopolymerization (day 0), therefore spanning day 1 to day 5.
